# Supplementary material for: Stacking Model‐Based Classifiers for Dealing With Multiple Sets of Noisy Labels
Source: Biom J. 2025 Mar 12;67(2):e70042. doi: 10.1002/bimj.70042 (PMC11898607; doi:10.1002/bimj.70042)
Supplement: Supplementary file 1 — Supporting Information [file BIMJ-67-e70042-s001.zip › density-based-ensemble-model-main/competitors_real_data/netlab-master/netlab-master/help/demgpard.htm]

Netlab Reference Manual demgpard


# demgpard

## Purpose

Demonstrate ARD using a Gaussian Process.

## Synopsis

```
demgpare
```

## Description

The data consists of three input variables `x1`, `x2` and
`x3`, and one target variable
`t`. The
target data is generated by computing `sin(2*pi*x1)` and adding Gaussian
noise, x2 is a copy of x1 with a higher level of added
noise, and x3 is sampled randomly from a Gaussian distribution.
A Gaussian Process, is
trained by optimising the hyperparameters
using the scaled conjugate gradient algorithm. The final values of the
hyperparameters show that the model successfully identifies the importance
of each input.

## See Also

`demgp`, `gp`, `gperr`, `gpfwd`, `gpgrad`, `gpinit`, `scg`

---

**Pages:**
Index


---

Copyright (c) Ian T Nabney (1996-9)
